# Workforce experience of the implementation of an advanced clinical practice framework in England: a mixed methods evaluation

**DOI:** 10.1186/s12960-020-00539-y

**Published:** 2020-12-03

**Authors:** Jessica Lawler, Katrina Maclaine, Alison Leary

**Affiliations:** 1 School of Health and Social Care, London South Bank University, 103 Borough Road, London, SE1 0AA UK; 2 University of South Eastern Norway, Drammen, Norway

**Keywords:** Health policy, Education and training, Health services administration and management

## Abstract

**Background:**

This study aims to understand how the implementation of the advanced clinical practice framework in England (2017) was experienced by the workforce to check assumptions for a national workforce modelling project. The advanced clinical practice framework was introduced in England in 2017 by Health Education England to clarify the role of advanced practice in the National Health Service.

**Methods:**

As part of a large-scale workforce modelling project, a self-completed questionnaire was distributed via the Association of Advanced Practice Educators UK aimed at those studying to be an Advanced Clinical Practitioner or who are practicing at this level in order to check assumptions. Semi-structured phone interviews were carried out with this same group. Questionnaires were summarised using descriptive statistics in Excel for categorical responses and interviews and survey free-text were analysed using thematic analysis in NVivo 10.

**Results:**

The questionnaire received over 500 respondents (ten times that expected) and 15 interviews were carried out. Advanced clinical practice was considered by many respondents the only viable clinical career progression. Respondents felt that employers were not clear about what practicing at this level involved or its future direction. 54% (287) thought that ‘ACP’ was the right job title for them. 19% (98) of respondents wanted their origin registered profession to be included in their title. Balancing advanced clinical practice education concurrently with a full-time role was challenging, participants underestimated the workload and expectations of employer’s training. There is an apparent dichotomy that has developed from the implementation of the 2017 framework: that of advanced clinical practice as an advanced level of practice within a profession, and that of Advanced Clinical Practitioner as a new generic role in the medical model.

**Conclusions:**

Efforts to establish further clarity and structure around advanced clinical practice are needed for both the individuals practising at this level and their employers. A robust evaluation of the introduction of this role should take place.

## Background

Advanced practice roles have existed in healthcare for many years. In the UK they were more formally established in the early 1990s [1]. Such roles have tended to evolve rather than be part of strategic workforce planning [2]. In other countries there has been a long history of advanced practice nursing, for example in the US and Australia, educational and regulatory frameworks protect the role and its identity [2]. In order to bring some clarity to these roles, in 2017 Health Education England (HEE) introduced a national multi-professional framework for advanced clinical practice in England. It was created to ensure national consistency and a definition of the level of practice [3]. It detailed the requirements for entry, the guidance and principles to adhere to and the career pathway. Advanced clinical practice was defined in 2017 as: “a level of practice characterised by a high degree of autonomy and complex decision making. This is underpinned by a master’s level award or equivalent that encompasses the four pillars of clinical practice, leadership and management, education and research, with demonstration of core capabilities and area specific clinical competence.” [3].

The concept of a multi-professional advanced clinical practice role is currently distinct to England and is relatively new. In this workforce advanced practice is not the role of nurses alone. Other professional groups such as pharmacists and Allied Health Professionals undertake a role known as an Advanced Clinical Practitioner [4–6]. Many calls have been made for a consistent approach to the development of this workforce across all occupational groups [7]. Prior to this, there had been some ambiguity over this level of practice and in particular the differences between advanced clinical practice and clinical specialisms [8,9]. Further, there was a lack of clarity in the expectation of the level of education needed. As initially most guidance in this area had been tailored for Advanced Nursing Practice, this may have resulted in variability in other professions without clear recognition [10,11]. A further factor adding to the confusion was the introduction of the term “Advanced Clinical Practitioner” by the Royal College of Emergency Medicine (RCEM) [12] and for the new masters level Apprenticeship [13]. RCEM introduced a specific role with a specific title that quickly became synonymous with advanced practice. This has resulted in confusion and as Mahase [14] has suggested, the ambiguity surrounding this level of practice has meant that instead of developing advanced practice in their registered profession, Advanced Clinical Practitioners (ACP) have been criticised for going into the jurisdiction of medicine, whether this be down to non-availability of resources or understaffing. As is clear in the introduction of any new role, navigation of existing professional identities is necessary. [15].

The skills and knowledge of advanced practitioners have in the past been described as “attributes” of the advanced practitioner role, but not as requirements [1]. Accreditation of the advanced practitioner role was also called for including robust role definition. The 2017 framework defines the core capabilities of advanced practice across four “pillars”: clinical practice, leadership and management, education and research [3]. These are described in different ways dependent upon the professional group, however, how competency can be assessed is not specified.

HEE’s 2017 national multi-professional framework for ACP states that the framework is “to be used as standard for healthcare providers, service providers, employers, service leads, education providers and health and care professionals practicing at, or aspiring to practice at, the level of advanced clinical practice.” [3]. HEE acknowledges the long-standing debate and differences between local and regional practice at advanced level. It further recognises that many titles are used for professionals who work at an advanced level and that “employers need to review their workforce in order to make sure that there is no misunderstanding by the public and the multi-disciplinary team.” Additionally, HEE’s framework suggests that employers need to evaluate, observe and address any concerns to ensure good governance of ACP. Whether or not these intentions have been met in the delivery of the framework is yet to be explored. Gaining current understandings from Advanced Clinical Practitioners themselves, could further our knowledge of the issues or inconsistencies.

The Nuffield Trust’s 2016 report “Reshaping the workforce to deliver the care patients need” commented on the challenges that a lack of an accepted definition of advanced practice posed [16]. Despite efforts to make skills for advanced practice clearer [4], different trusts reportedly have their own definitions and “many advanced roles are being developed in an ad-hoc fashion, within and across organisations” [16]. Whether the implementation of the HEE Framework in 2017 has to date has brought clarity or addressed and resolved these issues in England has not to date been explored. An evaluation of this sort on the implementation of the HEE 2017 framework has not been explored to date. Gaining knowledge of Advanced Clinical Practitioners’ views of the implementation of the HEE 2017 framework is important in understanding how the level of advanced clinical practice is carried out in different professions. It will enable consideration of further improvements that could be made to this level of practice, workforce policy going forward and identification of areas of success. Speaking directly with those involved currently will allow insight into this level of practice’s challenges, achievements and areas with room for improvement. It will also provide a base from which we can potentially increase the perceived value of the distinct contributions of each profession at this level. This analysis aims to provide insight into the implementation of the HEE ACP framework from the perspective of the workforce using secondary analysis of data collected from a commissioned workforce modelling project (Additional file 1).

## Methods

*Aim:* To gain insight into the workforce experience of the implementation of the 2017 HEE Advanced Practice Framework in England.

*Participants:* This is a secondary analysis of data from a HEE workforce modelling project. The underpinning workforce modelling project was commissioned by HEE to model another level of practice (enhanced practice) and this required insight into the implementation of the ACP Framework as part of the modelling. To do this several approaches were taken using soft systems modelling [17], which included extensive engagement with stakeholders. One of the groups was the advanced practice community who had reported anecdotally opportunities and challenges in the introduction for the ACP framework [3]. As modelling is iterative, it was necessary to understand these opportunities and challenges before and during the modelling of the enhanced practice workforce model [18].

Those working in or training for advanced practice roles were invited to take part in a self-completed questionnaire through an online link in an email to members of the Association of Advanced Practice Educators (AAPE) UK on behalf of HEE. The AAPE UK represents an influential collaborative network of Higher Education Institutions across the United Kingdom who are providers of advanced clinical practice programmes of education for interprofessional groups. This was also picked by the AAPE social media account (Twitter). A convenience sample was used.

*Materials and procedure:* Both the questionnaire and interview questions can be found in the appendices. This questionnaire was based on a previously validated questionnaire of NHS trusts and third sector workforce by Leary et al. [19]. At the end of the questionnaire, all participants were asked if they would be interested in being interviewed.

Participant information sheets were given to respondents that express an interest in interview, prior to taking part. Written consent was obtained by all those that took part in the questionnaire and the interview. The interviews were one-off semi-structured interviews that were audio recorded. Interviews were carried out by telephone by the first author and the duration was on average one hour.

There was no incentive to take part in interview. Field notes were taken during interview. Interviews were carried out post-questionnaire, enabling respondents to reflect on their questionnaire answers, in particular their free-text responses. Interview and survey questions can be found in the Additional file 1.

### Analysis

The questionnaire quantitative data were summarised using descriptive statistics in Excel. Interviews were transcribed and then Thematically Analysed [20] using NVivo™ (Version 10, QSR International) by the first and third authors. Both a deductive and an inductive approach to theme generation were used. Descriptive integration was used to merge the quantitative and qualitative data to make comparisons and for deeper understandings to emerge.

### Patient and public involvement

It was not appropriate to involve patients or the public in the design, conduct, reporting or dissemination plans of the research. Citizens were involved extensively in the modelling project.

## Results

The questionnaire was on Survey Monkey for 3 weeks and accessed 643 times. The questionnaire received 528 full responses and 115 partial responses (excluded from analyses). The original request target population for the modelling project was 50, with the final number of participants 528—ten times the original target. Fifteen interviews were carried out with ACPs from a variety of origin professions (profession prior to beginning ACP training and current regulated profession), from different regions of England. Nine interviewees were trainee ACPs and six were ACPs. Questionnaire respondents’ self-reported areas of clinical practice are detailed in Table 1.

**Table 1:** The questionnaire respondents’ areas of practice (*n* = 528)

Speciality	Respondents (%of total)
Acute gerontology	10 (2)
Acute medical (adult)	92 (17)
Acute medical (paediatric)	6 (1)
Acute mental health	8 (2)
Acute paediatric	19 (4)
Acute surgical/theatres	23 (4)
CAMHS	4 (1)
Community care	25 (5)
Community long term condition (e.g. respiratory)	11 (2)
Community mental health	16 (3)
Community paediatric	3 (1)
Critical care	28 (5)
Emergency Department (adult)	89 (17)
Emergency Department (adults and paediatrics)	7 (1)
Emergency Department (paediatrics)	9 (2)
Learning disability	2 (1)
Long term condition (e.g. cancer)	18 (3)
Midwifery	3 (1)
Neonatal	7 (1)
Other	19 (4)
Pre-hospital care	10 (2)
Primary care	104 (20)
Radiology	11 (2)
Radiotherapy	4 (1)
Total	528

**Fig. 1:**
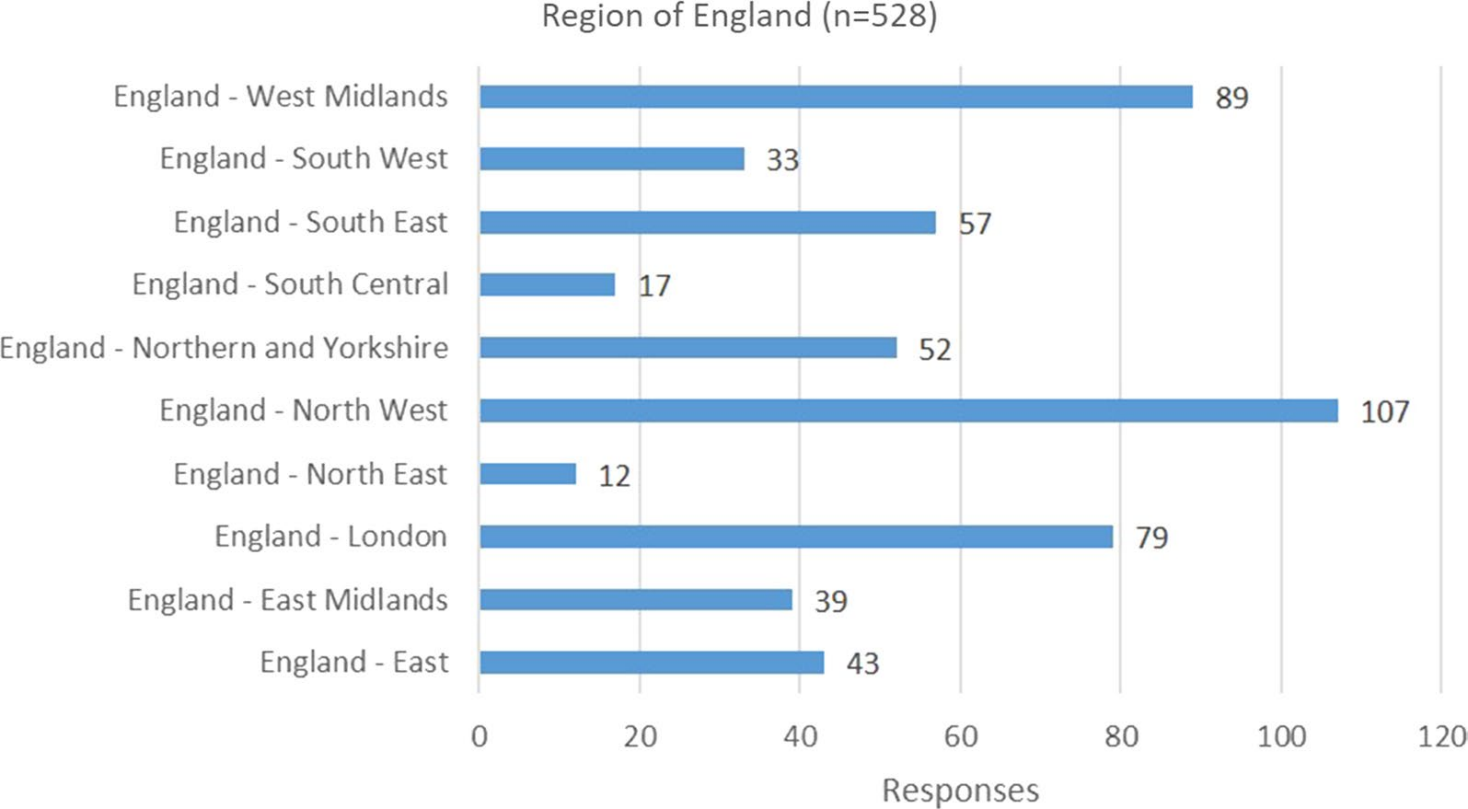
Area of the country in which the respondent practices

**Fig. 2:**
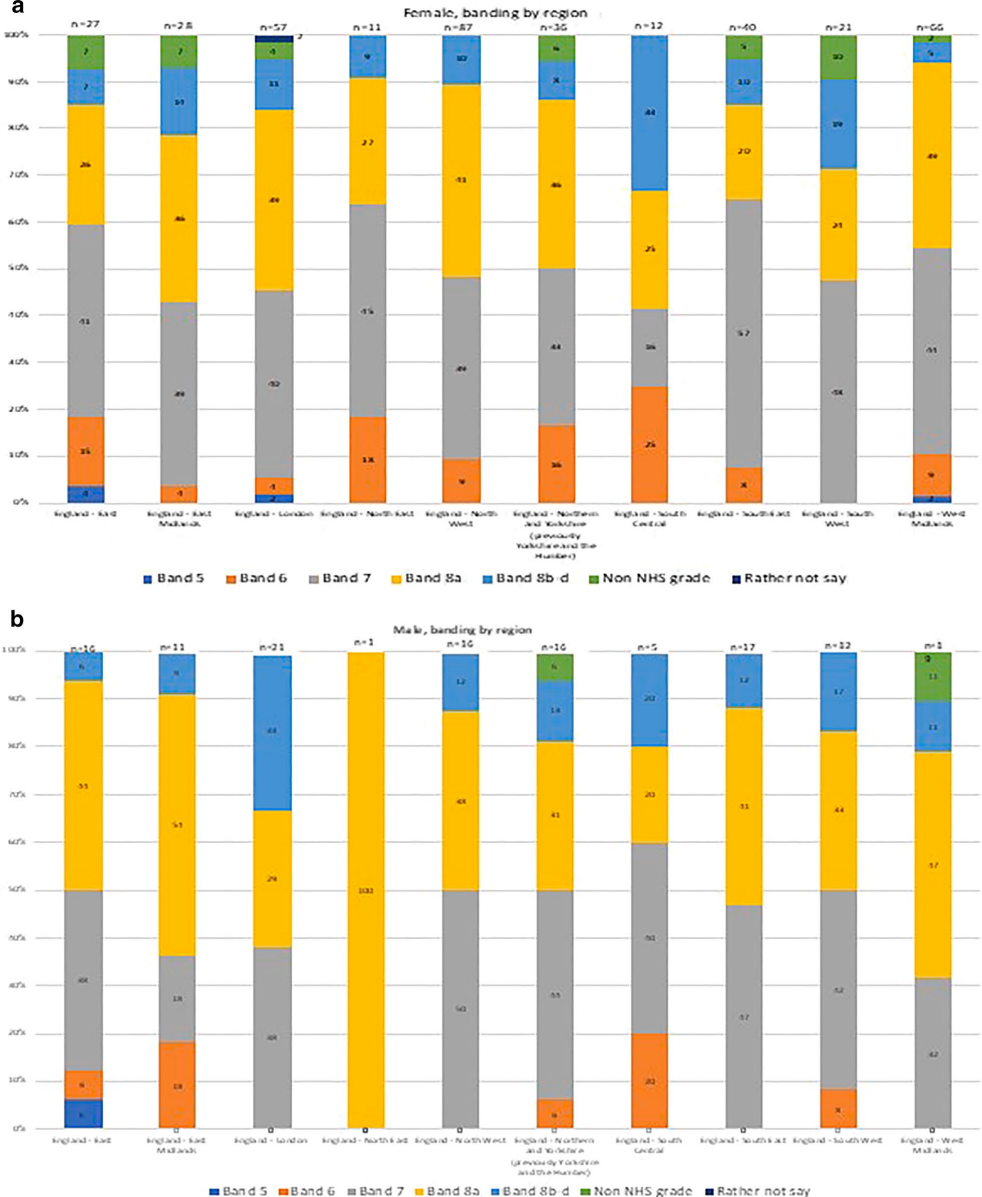
Respondent’s NHS banding by region, female and male

A third of questionnaire respondents’ titles were Advanced Clinical Practitioner, a third were trainee Advanced Clinical Practitioner, and the remaining third selected “other”. The respondents’ demography is detailed in Figs. 1 and 2.

**Fig. 3:**
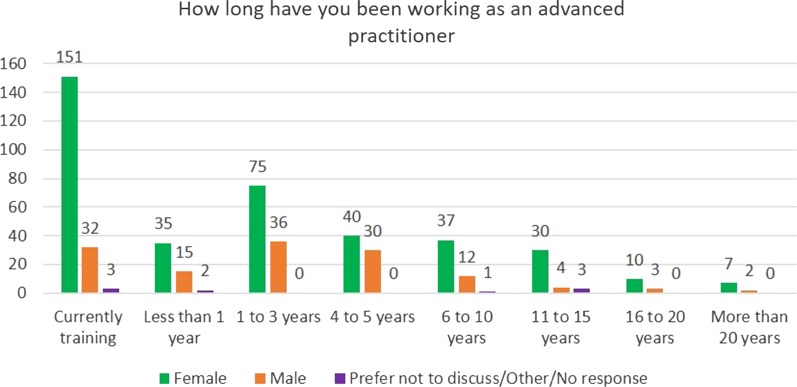
Current length of time working in advanced clinical practice

The largest groups of respondents were from Primary Care, Emergency Department (adult) and Acute Medical (adult). Respondent’s length of time as an advanced clinical practitioner is detailed in Fig. 3. The largest groups of respondent’s registered profession were nurse (365), paramedic (55), physiotherapist (40) and radiographer (diagnostic) (23).

In total, 35% of respondents were currently in education to become an ACP. This was the largest group of respondents followed by those who had been an ACP for 1–3 years (21%).

The majority of respondents (82%) were employed on a permanent NHS contract, 26% were rostered on a medical rota, and 66% were not. The National Health Service in England has a nationally agreed pay structure of banding. This starts at Band one and goes to Band 9. The majority of respondents were on Pay Band 7 (40%) or Pay Band 8a (36%). Only 3% of respondents were on a non-NHS grade. The smallest group of respondents (less than 1%) were pay band 5s, and there were 4 respondents in this group. Of those rostered on a medical rota, 9% (54) were paid locum medical rates if they undertook locum medical shifts.

There was variation within each group. For example out of the total 97 Emergency Department respondents 33% (*n* = 32) were trainees. Those that had been registered for 1–3 years was 28% (*n* = 27), and 2% (*n* = 2) had been registered for 16–20 years.

Data from the questionnaire are presented alongside the interview data to allow for comparison. Interview responses allow depth and further detail to some questionnaire responses. Questionnaire quotes are presented in italics and interview quotes in bold to provide further clarity between the data. The key themes and findings from interviews and free-text questionnaire answers are summarised in Box 1.

Box 1: Key findings

1. Advanced clinical practice was considered by 52% of the questionnaire respondents and all interviewees their only viable clinical progression option.
2. There is a lack of clarity around the structure of the ACP role, and its future.
3. Balancing training for advanced clinical practice with a full-time role was challenging.
4. 52% of questionnaire respondents thought their employer encouraged them to work across all four of the advanced practice “pillars”, 29% did not.

## Advanced clinical practitioner as a route for professional progression

Respondents suggested that there was a lack of opportunity available to progress clinically in their career apart from the ACP route. 52% of questionnaire respondents answered no, there were no other opportunities other than the ACP to progress clinically in their career, 32% responded yes, and 6% responded with not sure. Of those that answered no, 67% were nurses, 13% were paramedics, 11% were radiographers and 8% were physiotherapists. Of those that answered yes, 76% were nurses and 6% were paramedics.

Respondents indicated that alternative progression was only available in managerial roles with little if any patient contact, or that progressing clinically would result in having to drop a grade or not be promoted or paid for additional responsibilities.

Management was the only other option and I wanted to remain in clinical practice.

I don't know how you progress clinically if you don't do ACP. You progress managerially.

I had progressed as far as I could within my career path without becoming purely managerial.

Reasons for going into advanced clinical practice in the interviews ranged from a lack of other opportunities to progress, to advanced practice covering everything that they wanted.

The majority responded that they wanted to remain in clinical work, that their current role was not recognised as advanced and other progressions would lead them away from patient-facing work.

Because there was no progression where I was.—ACP in emergency medicine

When I looked into the role of the ACP, it just seemed to encompass the things that I wanted. So you were still very clinical, without all the managerial stuff to go with it.—Trainee ACP (surgery)

## Professional uncertainty

Questionnaire respondents were asked about where they see their professional aspirations and expectations. The dominant theme centred on the uncertainty of whether their employer will offer them a job as an ACP once they qualify, or if they will have to go elsewhere.

Not sure at present as our hospital do not have a clear pathway as to what is happening to us once we are qualified.

I cannot see any career progression within the ACP role locally. So either the status quo or a move to primary care. Banding would be unlikely to change. I feel stuck at this level now.

Interviewees also felt that employers did not understand what an advanced clinical practitioner would be able to do once qualified, and as a result there was a general lack of understanding particularly from immediate/middle managers.

Our trust doesn’t know what it wants its ACPs to do—Trainee ACP

Potentially all three of us could be sitting there with an ACP qualification, nobody really knowing what to do with us.—Community Matron (Adult Mental Health) ACP Trainee

Other questionnaire responses saw their career progression as aiming for a consultant role, moving more into research and a small number wanted to move completely out of healthcare.

Nothing to do with ACP as probably burnt out from pushing this boulder uphill

Many wanted to stay in the ACP role but with more acknowledgement and clarification around the role structure. 6% of respondents wanted to be involved in work to improve the ACP training experience for future ACPs.

Same but with a better set up. Perhaps helping others coming through to improve their experience over mine.

Interviewees also noted the lack of structure and plans set in place for when they started the role. Expectations of what they would be able to do added pressure to the ACPs to prove their worth and ensure that as pioneers of the role, they proved it was a success. The ACP role is seen as something novel despite advanced practice being established in UK healthcare for many years.

The executive boards didn’t know that we existed, there was no governance in place, nothing like that, so it was a bit of a challenge when we started.—ACP team leader

### Future of role

A recurring issue interviewees reported that although having no set plan in place when they began meant that they could contribute to shaping their future role, it was detracting from their current job and adding unnecessary stress and pressure.

It made me feel quite empowered that there was two of us shaping the future a little bit and how we were perceived and we were able to modify things like that so that was quite nice. But it made me feel like it detracted from what I was meant to be doing—ACP trauma and orthopaedics

I felt like there was a lot of added pressure to us because we were the first: we were trying to prove a point and make the role a success.—ACP trauma and orthopaedics

Others also noted that they thought they would be further in their career financially and clinically had they taken a different route, and the promises of what the ACP education would give them had not been fulfilled.

In terms of clinically…financially, I’m again going to have to be very honest with you, I feel I may have actually been further in my career if I had stayed in [specialism].—Trainee ACP

## Challenge of unpaid work

In the survey, 34% (179) of the total respondents did not work unpaid overtime. For trainees this this was 44% (78). Interviewees commented on the fact that clinical hours towards advanced clinical practice training had to be made up in their own time. Many were continuing with their origin profession while undertaking the education, and what respondents termed ACP hours had to be outside of these regular hours.

I have remained as my day to day hours as a Band Five and my day to day responsibilities are what you would expect of a Band Five and then I go in, in my own time, to do hours of practice for my course.—Emergency Department Trainee ACP

I still do my job as a [specialism] specialist nurse two days a week and that’s been my biggest stumbling block through my training really.—Trainee ACP

The expectation that they would have to continue their normal employment contract whilst completing the ACP course was in some cases not clear to respondents, and they found this a shock and an unexpected challenge.

What I didn’t realise was the expectation was that I would carry on doing the job that I was doing while I was doing my training.—Trainee ACP

This was described by multiple interviewees as the biggest challenge of their ACP experience. In one case an interviewee was undertaking ACP education in a clinical speciality completely different to their origin profession as this was the only area there was progression available, and they had to balance the work of two very different areas simultaneously, one of which was a new area of practice.

The demands of the course and, also, the clinical demands of having two jobs: that’s been really difficult—Trainee ACP (Surgery) (origin profession Diabetes nursing)

## Working across all four pillars

Interview respondents were asked whether they were encouraged or supported to work across all four pillars of advanced practice. Answers varied from some never having heard of the four pillars, to others covering all four pillars, and some saying there was a disproportionately large focus on the clinical pillar.

I don't know, to be honest no one really mentions the four pillars.—Trainee ACP (paramedic)

No one has ever brought up the four pillars.—Trainee ACP (paramedic)

There’s nothing in my contract to say it’s 70/30 clinical to non-clinical or 60/40 or 80/20: there’s nothing written down—ACP in Emergency Medicine

**Fig. 4:**
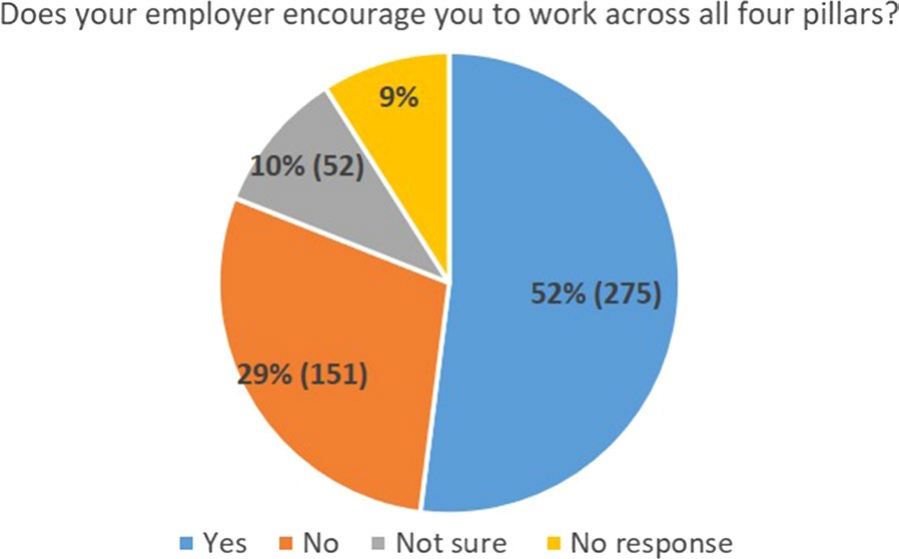
Responses to the question asking respondents if their employers encouraged working across all four pillars of advanced practice (education, research leadership and clinical practice)

29% (151) of questionnaire respondents responded that they did not think their employer encourages them to work across all four pillars, 10% (52) were not sure, and 52% (275) thought they did (Fig. 4).

## Advantages of the role

Respondents were asked about the best parts of their experience as an ACP. Many answers were on their ability to provide improved and more holistic patient care and to improve their knowledge and skills.

The best part is making a difference to the patients’ journey so it’s using the extended skills that I’ve learnt—Senior ACP (Emergency)

Understanding that the issues we have, we’re not isolated, everybody else is having similar struggles about how to apply advanced clinical practice in the different allied health professions—Trainee ACP

There is a lot of opportunity for us to demonstrate our aptitude and demonstrate the abilities of our role and shape it that way, from the inside—Trainee ACP (A&E)

## Issues and challenges

Interviewees were about their biggest challenges in their experiences of advanced clinical practice. Interview respondents all perceived that patients did not fully understand what being an Advanced Clinical Practitioner meant. Using the word ‘trainee’ when introducing themselves was also a source of contention, and sometimes caused resistance from patients.

I found it was quite a barrier to patients just saying the word ‘trainee’…I had one patient say I’m not having a trainee coming near me.—Trainee ACP

All interviewees also commented that they were required to explain their role to other colleagues, who they believed did not understand their role. This restricted them, and some felt that they were not able to carry out work to their full ability as a consequence.

The biggest challenge was making the workforce understand the role of advanced clinical practice—Trainee ACP

It’s also been extremely frustrating because I think we’re so restricted, it’s hard for people to get their head round it—Surgical Care Practitioner

Questionnaire respondents were asked whether they thought the “ACP” title accurately described their role. Of the 528 who answered, 54% (287) thought that ACP was the right job title for them. 19% (98) of respondents wanted their origin registered profession to be included in their title, and 3% (6) wanted their title to include their level of seniority as an ACP.

## Discussion

The 2017 Health Education England framework recommended employers review their workforce and to evaluate and address any concerns to improve understanding of advanced clinical practice for the public and for multi-disciplinary teams [3]. From the experiences of respondents and their discussion of challenges, understanding of advanced clinical practice varies between a level of practice and a role. Much of advanced clinical practitioner roles have developed without a set standard for each clinical background and therefore a large amount of discrepancy and variation exists. It is well documented that a lack of role clarity among stakeholders is a barrier to role implementation [21]. Among many other factors, introduction of a new role may interfere with pre-existing professional identities and hierarchies [15]^.^

Discrepancies between competencies, training and qualifications within the advanced title have created confusion within many professions. Regulatory and policy frameworks such as those in existence in other countries, to clarify and support this, are still much needed in defining the advanced clinical practitioner level in different areas, for employers and employees themselves [22] although they are now being developed [23,24]. Clarity and consistency around the ACP role is essential in ensuring avoidance of underutilisation [25]. The history of the development of the level of advanced practice in the UK is important to consider here. It has evolved across a systemic hierarchy of medical authority. Perceived advancement has been aligned with a medical ‘power’ base, rather than professional development in the original profession, and this seems to be reflected in the current status quo.

An idea of where the ACP role can take an individual in their career progression should be communicated openly and clearly. Within the 2017 HEE framework, the four “pillars” were portrayed as the pinnacle focus point of ACP’s “core capabilities” [3]. Clarity around the importance and priority of the four pillars of advanced practice is needed, as the responses surrounding the implementation of these suggested that employers stressed their importance differently. The 2017 HEE framework calls for employers to support, develop and facilitate ACPs to work across all four pillars [3]. The importance and added value of clinical, leadership, education, and research aspects on practice are widely reported [26,27].

There seems to be a dichotomy developing in terms of implementation of the HEE framework by employers between advanced clinical practice as a level of practice of the registered professional and advanced clinical practitioner as a novel omni professional role framed in the medical model as opposed to advancement of professional practice. This is unsurprising. 26% of respondents were rostered onto medical rotas and the views articulated of experienced practitioners becoming novice practitioners (trainees) in the medical model is likely to reflect the current deficit in the medical workforce and contribute to the workload as described by the participants. With other similar roles in the employment market such as physician associates there is a question about the sustainability of such an approach.

ACPs revealed that in some cases, employers expected advanced practice training to be carried out at the same time as their original job. This is not a new issue. Woods [9] noted that the most frequently identified inhibiting factor for ANPs was the expectation to develop in the ANP role “whilst being counted in the nursing numbers”. This difficulty has been identified in the past, yet in implementing advanced clinical practice it is evident that efforts have not been made to address the issue of added workload whilst developing the role. Role transitions, in particular the transition to nurse practitioner has been described as an ‘overwhelming’ process that is defined by straddling two identities and ‘transition shock’ is common [28–30]. This can result in a struggle to form an identity and feeling like an imposter [28].

Disengagement has commonly been reported, where continued connection to a prior role can prevent adjustment to the new role [28]. Furthermore, the transition from being experienced in a previous role to novice in a new role is a period of adjustment, and requires significant support [31]. As was identified in this evaluation, this was an area that respondents found challenging. Improved planning and system efforts are needed to address implementation issues that have been brought to light in previous introduction of new roles such as the APN^32^. There is a clear need for further clarity and structure to ACP training and role, for ACPs themselves, and for their employers. Further research into exploring specifics of what ACPs would have valued in their training and what support and structure they need within their current ACP role could provide huge benefit.

### Limitations

This evaluation provides insight into the opinions and experience of a group of advanced clinical practitioners in England. Therefore, the results of this evaluation are not generalisable to other populations or frameworks. With a small group of interviewees such as this, there is a risk that the participants’ views can be oversimplified. Convenience sampling was used. The previously validated questionnaire that this evaluation originated from was for a nurse population, although it has been used to model multi-professional workforces.

The questionnaire was emailed to 55 Association of Advanced Practice Educators (AAPE) UK members, and was also picked up by social media. Therefore, the number that this reached and an exact response rate is difficult to determine. There may be some bias from approaching only AAPE members. A strength of the study: the questionnaire received over 500 responses, ten times the target of 50. There may be some bias from approaching only AAPE UK members, however the purpose of this questionnaire was to elicit intelligence for a workforce modelling project. With a small group of interviewees such as this, there is a risk that the participants’ views can be oversimplified. This evaluation was part of a larger workforce modelling project and is incidental data. A formal evaluation of the role should take place given the findings.

## Conclusions

Efforts to establish further clarity and structure around advanced clinical practice are needed for both the individuals practising at this level and their employers. Issues around time for training and utilisation of the role also need consideration. Several far-reaching issues were raised in these data which were beyond the remit of the work, and so a robust evaluation of the introduction of this role should take place.

## Supplementary Information

**Additional file 1**. Interview and survey questions.

## Data Availability

Data are available from the author upon reasonable request.

## Abbreviations

ACP: Advanced clinical practice
AAPE: Association of Advanced Practice Educators
GDPR: General Data Protection Regulation
HEE: Health Education England
RECM: Royal College of Emergency Medicine
