# Supplementary material for: Workforce experience of the implementation of an advanced clinical practice framework in England: a mixed methods evaluation
Source: Hum Resour Health. 2020 Dec 3;18:96. doi: 10.1186/s12960-020-00539-y (PMC7713001; doi:10.1186/s12960-020-00539-y)
Supplement: Supplementary file 1 — Additional file 1. Interview and survey questions. [file 12960_2020_539_MOESM1_ESM.docx]

**Interview guide:**

1. Why did you choose to become an Advanced Clinical Practitioner?
2. What has your experience of the training for Advanced Clinical Practice been like for you?
3. Do you think your colleagues’ perceptions of your role accurately reflect your job?
4. What has worked well in your experience of being an Advanced Clinical Practitioner?
5. What challenges have you faced in your role?

**Questionnaire:**

1. Do you consent to take part in this questionnaire?

2. Please tell us which region of England you practise in.

3. Which sex do you most closely identify with?

4. What is your age?

5. What is your job title?

6. Does the ACP/trainee ACP title describe your role? If not what would your preferred title be?

7. Which clinical area do you practise in?

8. How long have you been working as an Advanced Clinical Practitioner?

9. What is your Registered Profession?

10. What kind of employment contract are you on?

11. Are you rostered on a medical rota?

12. Please tell us what Band you are currently on

13. How many hours per week are you contracted to work?

14. If you work unpaid overtime please estimate the average hours of unpaid overtime per week. When thinking about unpaid overtime please include working through meal breaks

15. If you do locum/agency work as part of a medical rota or other overtime are you paid

16. What qualifications do you hold or are currently studying for? If you have an overseas qualification please choose the closest equivalent from this list. Please tick all that apply

17. How is/was your training funded?

18. How much of your ACP training was funded by an employer or other organisation?

19. Would you recommend the ACP role to someone in your professional group?

20. Did you feel that there were other opportunities available to you to progress clinically in your career other than the ACP route?

21. Do you feel your origin profession has been valuable for your Advanced Clinical Practice role?

22. Do you have access to clinical supervision?

23. Do you think your line manager understands your role?

24. Do you think your employer encourages you to work across all four pillars (clinical practice, leadership and management, education and research)?

25. Do you think patients understand your role?

26. What challenges do you face in your role?

27. What has worked well in your experience of being an Advanced Clinical Practitioner?

28. What resources or support would have helped you in your progression to Advanced Clinical Practitioner?

29. Where do you see your career/what kind of work do you hope to be doing in five years?

30. If you are willing to share contact details please enter them here.
